# A Study on the Performance of Prestressed Concrete Containment with Carbon Fiber-Reinforced Polymer Tendons under Internal Pressure

**DOI:** 10.3390/ma16216883

**Published:** 2023-10-26

**Authors:** Xiaolan Pan, Aonan Tian, Lianpeng Zhang, Zhi Zheng

**Affiliations:** 1 College of Civil Engineering, Taiyuan University of Technology, Taiyuan 030024, China; 2 State Key Laboratory of Mechanical Behavior and System Safety of Traffic Engineering Structures, Shijiazhuang Tiedao University, Shijiazhuang 050043, China

**Keywords:** containment, failure mechanism, internal pressure, CFRP tendons, design recommendation

## Abstract

As the last barrier to preventing nuclear leakage, it is crucial to enhance the load-bearing capacity and cracking resistance of nuclear containment under internal pressure accidents. Currently, fiber-reinforced polymers are widely used in prestressing concrete structures because of their superior performance, but little research has been conducted on fiber-reinforced polymers in the field of nuclear power plants. In this paper, carbon fiber-reinforced polymer (CFRP) is used as a prestressing tendon material instead of traditional steel strands to study the damage mode of the new type of containment under internal pressure and the feasibility of using CFRP as prestressing tendons. In this study, a three-dimensional refinement model is established, employing ABAQUS 2020 software to analyze and quantify the pressure-bearing performance of nuclear containment with CFRP tendons and finally determine the reasonable range of CFRP tendons that can be used as a replacement. The research shows that the containment with CFRP tendons has an obvious strengthening effect in delaying the generation of cracks, restraining the speed of crack development, reducing the plastic damage of the steel liner, and improving the ultimate bearing capacity of the containment.

## 1. Introduction

Nuclear energy is a type of clean, safe, and economical energy. At present, nuclear power has become one of the world’s third major power supply pillars, together with hydropower and thermal power, due to its advantages of low resource consumption, low environmental impact, and strong supply capacity. Nuclear power technology has not only brought us huge dividends, but it has also brought potential threats. The nuclear leak at the Fukushima nuclear power plant in Japan sounded an alarm for people. As the last barrier to preventing nuclear leakage, nuclear power plant containment plays a vital role in ensuring nuclear power safety and preventing nuclear material leakage. Transforming the containments of nuclear power plants to have a better pressure-bearing capacity has become a great challenge in the scientific research community. Currently, many scholars are committed to studying the bearing capacity of the containment vessel. At Sandia National Laboratories, Horschel et al. [1] built a 1:6 large, reinforced concrete light water reactor containment to study its performance under overpressure. Hesseimer et al. [2,3] conducted pressure tests on the 1:4 scale model of prestressing concrete containment at Sandia National Laboratories and finally obtained the structural failure mechanism data. These experiments have obtained enough data to provide a sufficient basis for the study of the failure mechanism of the containment under internal pressure. However, such a large-scale test is time-consuming and requires a large amount of capital investment, so other researchers have focused on finite element simulations. Yan et al. [4] studied the mechanical behavior of prestressing concrete containment under overdesigned internal pressure through ABAQUS and revealed the failure mechanism of PCCV. Ahmad shokoohfar et al. [5] carried out a nonlinear analysis of prestressing concrete containment under internal pressure and high temperatures using a plastic damage model. Zhang et al. [6] analyzed the bearing capacity and safety margin of the containment under overpressure using the integral constructive model. Yan et al. [7] presented an enhanced finite element framework for the damage and failure analysis of PCCV. The framework incorporated element formulations, material models, and structural solution algorithms, greatly improving computational convergence performance and robustness. Jin et al. [8] studied the failure probability and vulnerability of nuclear containment under internal pressure using the probability method and sampling calculation with ABAQUS. Zhou et al. [9] also conducted a reliability analysis on the CPR1000 PWR model using a stratified sampling method. Ren et al. [10] considered more than one failure mode in the vulnerability evaluation and used an equivalent extreme value event combined with PDEM to obtain the failure probability and vulnerability curve of PCCV. However, although the above-mentioned studies were conducted to research the ultimate bearing capacity of the containment, how to improve the crack resistance and pressure-bearing performance of the containment is still one of the many challenging problems.

In the past few decades, it has been found that the use of fiber as a reinforcing material in concrete or rebar can effectively improve the crack resistance of reinforced concrete structures. In fact, the application of FRP tendons in concrete structures has thrived. Many scholars have conducted experiments on the interaction between FRP tendons and concrete and have drawn reliable conclusions. Researchers [11,12,13] pointed out that the axial strain of longitudinal FRP tendons in concrete columns can be used to accurately predict their contribution to the axial load borne by the columns. Other scholars [14,15,16] made GFRP tendons with the compressive strength being 35% of its tensile strength and then put forward the calculation formula for the axial compression bearing capacity. When GFRP tendons were replaced with equal area reinforcement, the axial compression bearing capacity would decrease, but the ductility was close [17]. The greater the ratio of the longitudinal reinforcement, the greater the ductility and bearing capacity of the specimen. Ye et al. [18] investigated the shear performance of CFRP-reinforced concrete beams and showed that changing the reinforcement distribution could increase the shear load capacity of the beams and increase the load corresponding to the maximum allowable crack width. Prabin et al. [19] conducted four-point bending tests on different types of FRP-reinforced beams, and the study showed that the load deflection curve was linear for all beams before the first crack appeared. The CFRP-reinforced concrete beams performed better than the GFRP-reinforced and BFRP-reinforced concrete beams under the same conditions. Muhammad Masood Raft et al. [20] analyzed the flexural properties of CFRP-reinforced concrete beams. The test results showed that the crack development of CFRP-reinforced concrete beams was similar to that of reinforced concrete beams. The difference was that the reinforced concrete beams exhibited reinforcement yielding when damaged, while the CFRP-reinforced concrete beams underwent concrete crushing when damaged. In corrosion studies of GFRP tendons and CFRP tendons, Zhang et al. [21] found that the tensile strength deterioration of CFRP tendons after immersion in acid and alkaline salt media was much less than that of GFRP tendons. Micelli et al. [22] also concluded that the mechanical properties, alkali resistance, and aging resistance of CFRP tendons were better than those of GFRP tendons by performing alkali resistance and aging tests on both CFRP and GFRP fibers. Hong et al. [23] concluded that there was no significant change in the strength of CFRP tendons in 120 h aging tests in the alkaline environment, while both GFRP and AFRP tendons showed relatively large strength depreciation. Yong et al. [24] concluded that the alternating wet and dry environments had little effect on the tensile strength of CFRP tendons by simulating seawater immersion of CFRP tendons.

However, most of the tests and research on FRP composite strengthening conventional structures and members at present are focused on beams, plates, and columns, while research on FRP strengthening or replacement for special structures such as nuclear power plant containment is still scarce. Although Homam and Sheikh [25] conducted the durability test of FRP reinforcement in the nuclear power plant environment and proposed the potential application prospect of FRP reinforcement in the nuclear power field, the comprehensive performance of nuclear containment by the addition of FRP tendons still needs deeper investigation. Taking the containment of the third-generation advanced nuclear power plant in China as the research object, this paper uses ABAQUS 2020 finite element software to realize the refined modeling of prestressing concrete containment. Then, the conventional prestressing steel strands are replaced by prestressing CFRP tendons. After that, the pressure-bearing performance, the evolution of concrete cracks, and the safety performance indices of the new containment are fully discussed and compared with the conventional prestressing concrete containment. Finally, a reasonable range of CFRP tendons for the new containment under normal use conditions is finally recommended.

## 2. Finite Element Model

### 2.1. Containment Geometry

**Figure 1:**
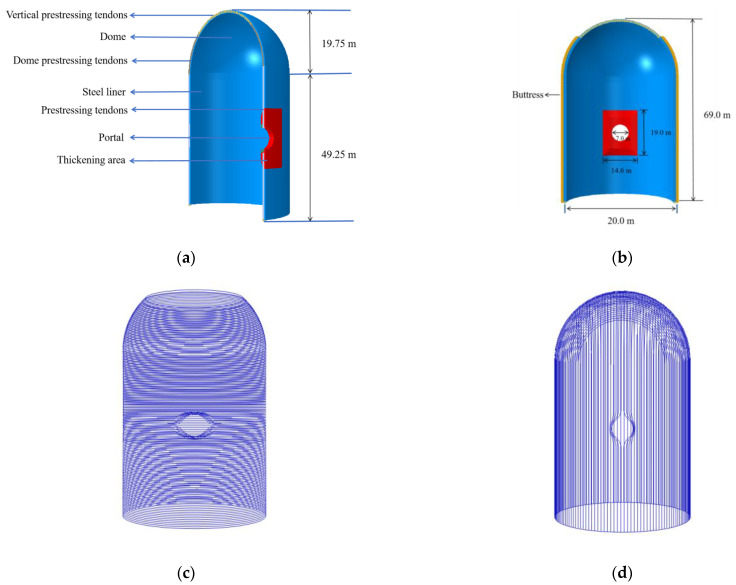
Geometry of the containment: (**a**) structural drawing of side section; (**b**) structural drawing of front section; (**c**) horizontal prestressing tendons; (**d**) vertical prestressing tendons.

The model is established with reference to China’s third-generation nuclear power plant. The main structure of the containment vessel is composed of a semicircular dome, a concrete cylinder, and buttresses, as shown in Figure 1. From the outside to the inside, the structure is composed of concrete, an ordinary reinforcement layer (i.e., rebar layer), prestressing tendons, and a steel liner. The height of the containment is 69 m; the radius of curvature of the upper semicircular dome is 19.75 m; and the wall thickness is 1 m. The height of the lower concrete cylinder is 49.25 m; the inner diameter is 20 m; and the wall thickness is 1.1 m. A rectangular, thickened area with a width of 14.6 m and a height of 19 m is set at the height of 25.6 m, and an equipment hole with a diameter of 7 m is left in the middle. A 6 mm thick steel liner is embedded inside the containment, which is closely connected with the concrete through rivets. Two buttresses are symmetrically arranged at 0° and 180° outside the containment for tensioning and anchoring of prestressing tendons. The prestressing concrete containment shell is constructed using the post-tensioning method. The tensioning system consists of 330 prestressing tendons, of which 190 circumferential prestressing tendons are anchored to the buttresses with a reinforcement ratio of 1.55% and 140 longitudinal prestressing tendons are anchored to the bottom slab with a reinforcement ratio of 0.55%. The double-layered reinforcement was arranged vertically and horizontally with a circumferential spacing of 0.133 m and a vertical spacing of 0.146 m, fully embedded in the concrete.

### 2.2. Establishment of Numerical Model

The finite element software ABAQUS 2020 is used to model the containment. The concrete is divided into meshes by a three-dimensional eight-node solid reduced integral element (C3D8R), and the steel liner is represented by a four-nodes reduced integral shell element (S4R), which is constrained to the inner surface of the containment by node displacement. The prestressing tendons and CFRP tendons are meshed by two node truss elements (T3D2), without considering the relative slip between the prestressing tendons and the surrounding concrete. This is reasonable because the “U” type and horizontal prestressing tendons are perfectly fixed to the base and buttress columns, respectively, according to the design of the containment, which can make the concrete and the prestressing tendons jointly bear the internal pressure load almost without relative slip. The ordinary reinforcement layer is represented by surface elements (SFM3D4R) and is completely embedded in the concrete. Chakraborty et al. [26] found that the presence of the base slab did not significantly influence the behavior of the containment structure. Therefore, the base slab is not explicitly modeled in this study.

The mesh division of the containment will have a great impact on the calculation results. Too-fine mesh will lead to low calculation efficiency, and not enough fine mesh will lead to inaccurate calculation results. A detailed sensitivity analysis for the effects of the mesh size on the numerical analysis results was conducted by Zheng et al. [27]. Herein, the recommended mesh size with the 0.8 m mesh is determined in this study. Additionally, the comparison analysis of comparable containment under cyclic static load and increasing internal pressure load between numerical and test results was performed in our previous study [28]. The above numerical method can ensure the fidelity of the numerical model.

### 2.3. Material Constitutive Model

#### 2.3.1. Concrete Material Properties

**Figure 2:**
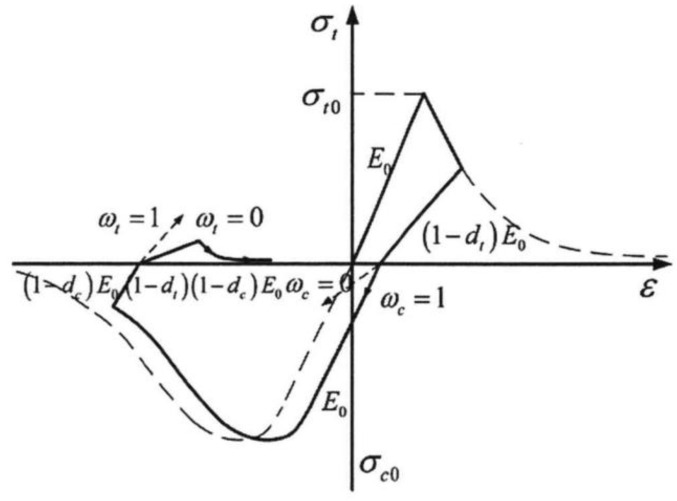
Concrete stress–strain relationship.

The concrete adopts the concrete plastic damage (CDP) model [29], which is broadly used to simulate isotropic elastoplastic materials. The basic material parameters, damage factors, and stress–strain relationship are determined according to the Chinese code (GB50010-2002) [30]. The material properties of concrete are shown in Table 1. The stress–strain relationship curve of concrete under compression and tension conforms to the Chinese code (GB50010-2002) [30], as shown in Figure 2.

The stress–strain curve of concrete under uniaxial compression can be determined by the following formula:

(1) $$\begin{array}{c} \sigma_{c}= \end{array}\left\{\begin{array}{c} \alpha_{a}x_{c}+\left(3-2\alpha_{a}\right)x_{c}{}^{2}+\left(\alpha_{a}-2\right)x_{c}{}^{3},\begin{array}{cc} & x_{c}\leq 1 \end{array}\\ \frac{x_{c}}{\alpha_{d}{\left(x_{c}-1\right)}^{2}+x_{c}},\begin{array}{cc} & x_{c}>1 \end{array} \end{array}\right.$$

(2) $$x_{c}=\frac{\varepsilon_{c}}{\varepsilon_{c,r}}$$

(3) $$d_{c}=1-\sqrt{\frac{\sigma_{c}}{E_{0}\varepsilon_{c}}}$$

(4) $$\left\{\begin{array}{c} {\tilde{\varepsilon }}_{c}^{\mathrm{in}}=\varepsilon_{c}-\frac{\sigma_{c}}{E_{0}}\\ {\tilde{\varepsilon }}_{c}^{\mathrm{pl}}={\tilde{\varepsilon }}_{c}^{\mathrm{in}}-\frac{d_{c}}{1-d_{c}}\frac{\sigma_{c}}{E_{0}} \end{array}\right.$$

where $\alpha_{a}$ and $\alpha_{d}$ represents the coefficients of the rising and falling sections of the uniaxial compression stress–strain relationship curve of concrete; $\varepsilon_{c}$ and $\varepsilon_{c,r}$ denote compressive strain and peak compressive strain of concrete, respectively; $d_{c}$ represents a damage parameter [31]; $\sigma_{c}$ represents compressive stress of concrete; $E_{0}$ denotes an elastic modulus of concrete; ${\tilde{\varepsilon }}_{c}^{\mathrm{in}}$ represents the relative inelastic strain; ${\tilde{\varepsilon }}_{c}^{\mathrm{pl}}$ represents the compression equivalent plastic strain [29].

The stress–strain relationship curve of concrete under uniaxial tension can be determined using the following formula:

(5) $$\begin{array}{c} \sigma_{t}= \end{array}\left\{\begin{array}{c} 1.2x_{t}-0.2x_{t}{}^{6},\begin{array}{cc} & x_{t}\leq 1 \end{array}\\ \frac{x_{t}}{\alpha_{t}{\left(x_{t}-1\right)}^{1.7}+x_{t}},\begin{array}{cc} & x_{t}>1 \end{array} \end{array}\right.$$

(6) $$x_{t}=\frac{\varepsilon_{t}}{\varepsilon_{t,r}}$$

(7) $$d_{t}=1-\sqrt{\frac{\sigma_{t}}{E_{0}\varepsilon_{t}}}$$

(8) $$\left\{\begin{array}{c} {\tilde{\varepsilon }}_{t}^{\mathrm{ck}}=\varepsilon_{t}-\frac{\sigma_{t}}{E_{0}}\\ {\tilde{\varepsilon }}_{t}^{\mathrm{pl}}={\tilde{\varepsilon }}_{t}^{\mathrm{ck}}-\frac{d_{t}}{1-d_{t}}\frac{\sigma_{t}}{E_{0}} \end{array}\right.$$

where $\alpha_{t}$ represents the coefficients of the falling sections of the uniaxial tensile stress–strain relationship curve; $\varepsilon_{t}$ and $\varepsilon_{t,r}$ denote compressive strain and peak compressive strain, respectively; $d_{t}$ represents a damage parameter; $\sigma_{t}$ represents tensile stress; ${\tilde{\varepsilon }}_{t}^{\mathrm{ck}}$ represents the relative inelastic strain; ${\tilde{\varepsilon }}_{t}^{\mathrm{pl}}$ represents the tensile equivalent plastic strain.

#### 2.3.2. FRP Material Properties

FRP tendons are made of continuous fibers (such as glass fiber and carbon fiber) glued together using base materials (such as polyamide resin, polyethylene resin, and epoxy resin), and then extruded and drawn by special molds.

The common fiber materials used for FRP tendons are generally glass fiber-reinforced plastic (GFRP), carbon fiber-reinforced plastic (CFRP), aramid fiber-reinforced plastic (AFRP), etc. Owing to their great advantages such as light weight, high tensile strength, strong corrosion resistance, strong material binding force, and strong magnetic wave permeability, FRP tendons have been widely used in structural reinforcement and engineering transformation in recent decades [32,33].

The stress–strain curve of FRP tendons is linear without an obvious yield stage, and the failure mode is brittle failure [33]. In the primary stage of stretching, the resin is mainly used to carry the tensile force. After the resin is gradually destroyed, the fiber bundle mainly bears the tensile force. When the ultimate load is reached, the fiber bundle breaks. To ensure that the structure has enough strength capacity reserve, when FRP tendons are applied in engineering, 75–80% of their ultimate tensile strength is taken as the design value, which is called nominal yield strength, in view of their high tensile strength.

The FRP stress–strain relationship is as follows [34]:

(9) $$\sigma_{f}=E_{f}\varepsilon_{f}$$

where $\sigma_{f}$ represents stress; $E_{f}$ represents the elastic modulus; $\varepsilon_{f}$ represents strain.

**Figure 3:**
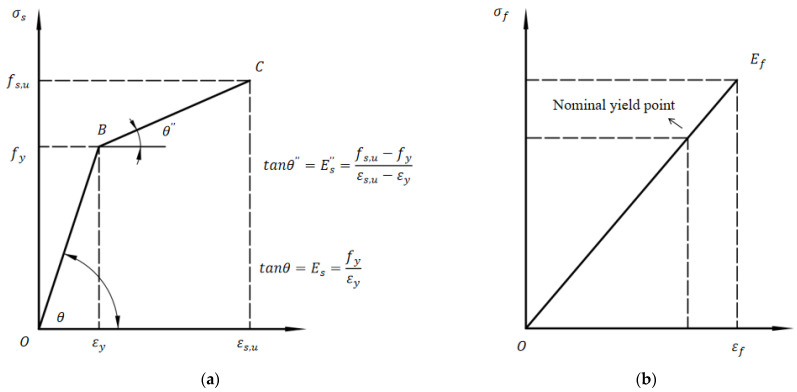
Stress–strain relationship; (**a**) stress–strain relationship of rebar; (**b**) stress–strain relationship of FRP tendons.

The bilinear model is used for ordinary reinforcement, and the linear elastic model is used for prestressing FRP tendons [35]. The typical stress–strain relationship curves of ordinary reinforcement (i.e., rebar) and FRP tendons are given in Figure 3.

Table 2 summarizes the material properties of steel and different FRP materials. The FRP tendons differ from steel strands in that the resin in the FRP tendons controls its mechanical properties in the transverse direction, while the fibers control their mechanical properties in the longitudinal direction. The ratio of resin to fiber will change the mechanical properties of the fiber tendons. In Table 2, as compared with other FRP materials, the performance of CFRP tendons is better because of their larger ultimate tensile strength and elastic modulus, which are close enough to the steel material. Therefore, the CFRP tendon is chosen as the object of the study. As seen in Table 2, the ultimate tensile strength of CFRP tendons ranges from 600 MPa to 3700 MPa. To fully develop the tensile capacity of CFRP tendons, the ultimate tensile strength of CFRP tendons ranging from 2500 MPa to 3500 MPa is considered. Since the CFRP tendon is a brittle material, in order to prevent its brittle damage during use, 80% of its ultimate tensile strength is generally taken as the nominal yield strength in engineering. Thus, its actual yield strength ranges from 2000 MPa to 2800 MPa. Generally speaking, the tensile strength of CFRP tendons is much higher than that of rebars, and the difference in elastic modulus is also great. In order to avoid the adverse effects of these two kinds of rebars when working together, it is better to choose CFRP tendons with an elastic modulus lower than steel strands [36]. Herein, the determination of its modulus of elasticity is in the range of 125 GPa~200 GPa.

To have a comprehensive understanding of the feasibility and applicability of CFRP tendons adopted for nuclear containment, different combinations of yield strength and modulus of elasticity are considered, as these two variables can have a significant effect on the pressure-bearing performance of the nuclear containment. Therefore, this paper sets group A for conventional steel strands, while groups B to F are assigned for CFRP tendons, as exhibited in Table 3. Specifically, groups B-1 to B-4 are represented for CFRP tendons with the constant yield strength of 2000 MPa and different elastic modulus from 125 GPa to 200 GPa with an interval of 25 GPa. Groups C-1 to C-4 are characterized for CFRP tendons with the constant yield strength of 2200 MPa and different elastic modulus from 125 GPa to 200 GPa with an interval of 25 GPa. Groups D-1 to D-4 are denoted for CFRP tendons with the constant yield strength of 2400 MPa and different elastic modulus from 125 GPa to 200 GPa with an interval of 25 GPa. Groups E-1 to E-4 are represented for CFRP tendons with the constant yield strength of 2600 MPa and different elastic modulus from 125 GPa to 200 GPa with an interval of 25 GPa. Groups F-1 to F-4 are assigned for CFRP tendons with the constant yield strength of 2800 MPa and different elastic modulus from 125 GPa to 200 GPa with an interval of 25 GPa.

## 3. Analysis Method and Theory

### 3.1. Loading Step and Boundary Conditions

Three analysis steps are applied in ABAQUS. The first step is to apply a gravity effect to the containment. Subsequently, the second step is to exert prestress. The loss of prestress is generally categorized into short-term and long-term prestress loss. The short-term prestressing loss mainly comprises the anchor deformation due to the tension end and the retraction of prestressed tendons $\sigma_{l1}$, and the friction between the prestressed tendons and the duct $\sigma_{l2}$. The prestress loss of long term is mainly caused by the stress relaxation of prestressed tendons $\sigma_{l3}$ and the shrinkage and creep of concrete $\sigma_{l4}$. According to the Chinese Code (GB50010-2002) [30], the above prestress losses are calculated and predicted. The expressions for the calculation of the prestress loss are given in Equations (10)–(13).

(10) $$\sigma_{l1}=\frac{a}{l}E_{s}$$

(11) $$\sigma_{l2}=\sigma_{\mathrm{con}}(1-\frac{1}{e^{kx+\mu \theta }})$$

(12) $$\sigma_{l3}=0.2(\frac{\sigma_{\mathrm{con}}}{f_{\mathrm{ptk}}}-0.575)\sigma_{\mathrm{con}}$$

(13) $$\sigma_{l4}=\frac{0.9\alpha_{p}\sigma_{pc}\varphi_{\infty }+E_{s}\varepsilon_{{}_{\infty }}}{1+15\rho }$$

where *a* includes the anchorage deformation of tension end and retraction of prestressed tendons; *l* represents the length between the anchorage end and the tension end; *E_s_* is the elastic modulus of prestressed tendons; $\sigma_{\mathrm{con}}$ is the control stress for prestressing; *k* is the friction coefficient of partial diversion per meter in the duct; *x* measures the plane distance from the calculated between the prestressed tendons and the duct; θ represents the tangent angle of the calculated cross section in the duct curve; $\alpha_{p}$ represents the ratio of elastic modulus of tendons to the value of concrete; $\sigma_{\mathrm{pc}}$ is the normal stress of concrete in compression at the point of composition of forces under the tensile area of tendons; ρ is the total ratio of reinforcement; $\varphi_{\infty }$ is the ultimate value for coefficient of shrinkage of concrete; $\varepsilon_{\infty }$ is the ultimate value of concrete shrinkage strain. The prestress loss in the vertical and circumferential directions of the steel strands and CFRP tendons are calculated based on the above equation, as shown in Table 4.

Lastly, the third step is to apply internal pressure. The exertion of prestress is realized by the cooling method, and the formula is as follows:

(14) $$\Delta T=\frac{\Delta \sigma }{\lambda E}$$

where ΔT is the cooling temperature of prestressing tendons; Δσ is the tension control stress, wherein the control stress of the steel strand is 0.8 $f_{\mathrm{ptk}}$ ($f_{\mathrm{ptk}}$ is the standard value of the ultimate tensile strength of the steel strand), and the control stress of the prestressing CFRP tendons is 0.65 $f_{\mathrm{ptk}}$ (refer to Chinese code GB50608-2010 [37]); λ is the linear expansion coefficient of the prestressing tendons; E is the elastic modulus of the prestressing tendons.

In fact, the bottom of the containment is fully embedded in the base slab, so the bottom of the containment is constrained in x, y, and z directions during modeling.

### 3.2. Containment Failure Criterion

The damage modes of containment are classified as functional failure and structural failure. Functional failure is defined as the loss of the containment’s function to prevent leakage. Structural failure is defined in terms of the structural ultimate state of the containment and is generally considered to be the overall deformation and strain of the structure exceeding the corresponding limit values. In this paper, the damage criterion for concrete containment suggested by the literature [40] is adopted, which can be summarized as follows: (1) steel liner reaches the state of yielding or tearing; (2) prestressing tendons reach the yield state.

### 3.3. Equivalent Stiffness Theory

Since there are few design examples of FRP tendons at present, this paper adopts the equivalent stiffness design principle proposed by Zhang and Ou [41] to design tendons for the containment. Based on the equivalent stiffness theory, FRP tendons with a lower elastic modulus and larger size shall be used to replace the rebars with smaller size and rigidity equal to FRP tendons. Currently, this criterion has been used for the corresponding calculation, analysis, and design of members and structures. Based on this criterion, it is only necessary to appropriately increase the cross-sectional area of CFRP tendons and increase the reinforcement rate according to the characteristics of CFRP tendons to ensure that it has the same stiffness as the original enclosure structure. More importantly, as CFRP tendons have greater reserves in strength than steel strands, the ultimate bearing capacity of FRP tendons’ structure beyond the normal using state is further reserved and improved. The dimensional information of the steel strands and CFRP tendons is shown in Table 5.

## 4. Analysis Results

### 4.1. Crack Evolution of Concrete

As shown in Table 6, at the initial stage of internal pressure application, the whole containment is in the elastic stage and the concrete is basically free of cracks. When the internal pressure increased to 0.8 MPa, small cracks appeared at the equipment hatch of the conventional containment. When the internal pressure increased to 1.1 MPa, the began to appear around the equipment hatch and cracks on the upper and lower sides increased significantly. In addition, small cracks appeared at the top of the dome and the bottom of the cylinder. When the internal pressure increased to 1.5 MPa, the cracks almost covered the whole concrete cylinder, and the damage near the equipment hatch was the most serious. In addition, a deep “X”-type inclined crack was clearly seen. When the ultimate internal pressure was reached, the containment completely failed and lost its working capacity.

The elastic modulus of prestressing tendons was taken as a fixed value and the yield strength was taken as a variable to analyze the results. When the internal pressure was 1.1 MPa, the failure characteristics of the 2200 MPa CFRP containment were the same as those of the conventional containment, and obvious cracks appeared at the equipment hatch, the top of the dome, and the bottom of the concrete cylinder. However, there were only small cracks at the equipment hatch for the containment with 2400 MPa and 2600 MPa CFRP tendons. When the internal pressure increased to 1.5 MPa, the damage degree of the containment with 2400 MPa and 2600 MPa CFRP was obviously less than that of the conventional containment. It can be seen that only arc cracks appeared near the equipment hatch and the dome top, and most part of the cylinder remained in a good condition. When the ultimate internal pressure was the containment with 2600 MPa CFRP tendons had the least damage, and the containment was not completely damaged.

Considering the yield strength as a constant value, the effect of elastic modulus on the pressure-bearing performance of the containment was also analyzed. When the yield strength of prestressing tendons remained the same, the damage characteristics of containment were not different. It can be seen that the influence of elastic modulus on the crack evolution was less than the impact of the yield strength of prestressing tendons on the crack evolution of the containment.

**Figure 4:**
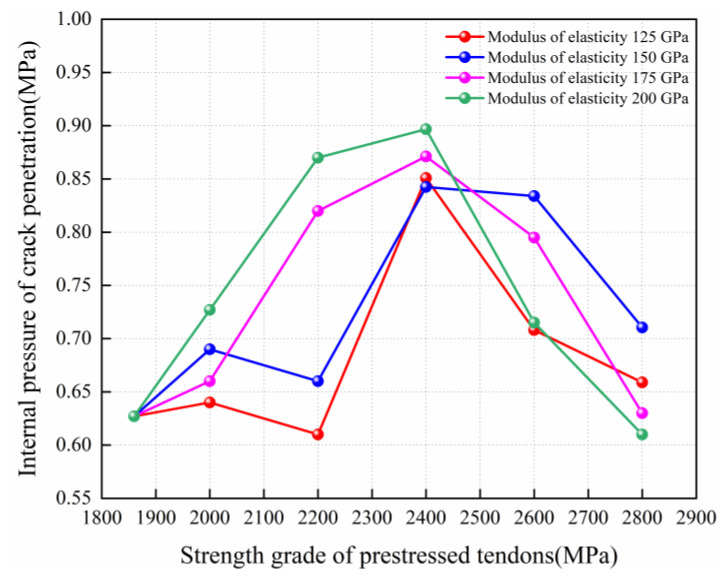
Comparison of internal pressure capacity for concrete crack penetration with CFRP tendons having different yield strength grades and elastic modulus.

Concrete cracking is critical to the assessment of containment leakage rates. Figure 4 summarizes the internal pressure capacity corresponding to the concrete crack penetration for different types of containment. It was found that the crack penetration phenomenon occurs when the internal pressure of the conventional containment reaches 0.627 MPa. When the elastic modulus of CFRP tendons was 150 GPa, the 2200 MPa, 2400 MPa, and 2600 MPa CFRP tendons, respectively, increased the internal pressure value of crack penetration to 0.661 MPa, 0.843 MPa, and 0.834 MPa. Compared with conventional containment, the internal pressure value for the crack penetration state increased by 5.3%, 34.37%, and 33.1%, respectively. When the elastic modulus of the CFRP tendons was 200 GPa, the 2200 MPa, 2400 MPa, and 2600 MPa CFRP tendons, respectively, increased the internal pressure value of crack penetration to 0.871 MPa, 0.897 MPa, and 0.715 MPa, resulting in 38.8%, 43.01%, and 14.1% higher results than those of conventional containment. The most significant increase in the internal pressure capacity for crack penetration was for the replacement scheme using 2400 MPa CFRP tendons. When the yield strength of CFRP tendons reached 2600 MPa or more, the enhancement effect experienced a significant decrease. This is because the larger yield strength of the CFRP tendons may induce the equipment hatch to be crushed at the initial stage of internal pressure application, which can have a disadvantageous effect on the subsequent pressure bearing for the containment.

### 4.2. Strain of the Steel Liner

**Figure 5:**
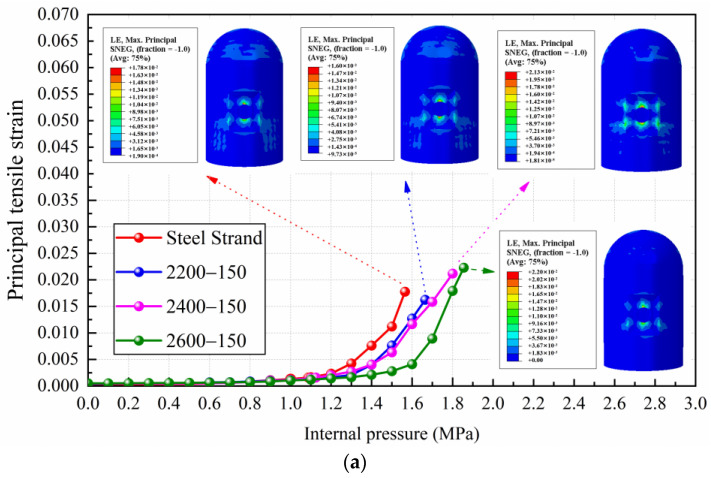

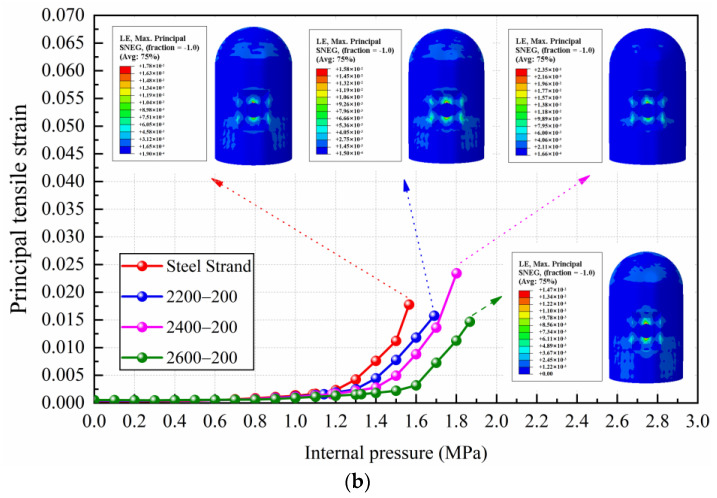
The results of principal tensile strain of steel liner for conventional and CFRP containments: (**a**) comparison of steel strand and CFRP tendons with 150 GPa elastic modulus and different yield strength levels; (**b**) comparison of steel strand and CFRP tendons with 200 GPa elastic modulus and different yield strength levels.

The yield of steel liner is another important index used to judge the failure of containments. The results of the principal tensile strain of steel liner for conventional and CFRP containments are illustrated in Figure 5. As shown in Figure 5, before the internal pressure of 1.1 MPa, the principal tensile strain value of the steel liner for various types of containment increases very slowly with the increasing internal pressure. After the internal pressure reaches 1.1 MPa, the steel liner generally begins to yield, and the principal tensile strain increases sharply with the increasing internal pressure. It can be seen that after replacing CFRP tendons, the rising trend of the principal tensile strain of the steel liner obviously slowed down. The steel liner of conventional containment will yield when the internal pressure value is 1.087 MPa, and when replacing the prestressing tendons with CFRP tendons with a modulus of elasticity of 150 GPa and yield strengths of 2200 MPa to 2600 MPa, the internal pressure values corresponding to the yielding state for the steel liner are delayed to 1.117 MPa, 1.125 MPa, and 1.348 MPa, respectively. When the containment uses CFRP tendons with a modulus of elasticity of 200 GPa and yield strengths of 2200 MPa to 2600 MPa, the internal pressure values for the steel liner’s yielding state are delayed to 1.142 MPa, 1.181 MPa, and 1.287 MPa, respectively. Among them, the CFRP tendons with the yielding strength of 2600 MPa have the greatest effect on the internal pressure capacity for the steel liner at yielding, which can be increased by 17.6% and 24.1% for the elastic modulus of 150 GPa and 200 GPa, respectively. 

When the conventional containment reaches the ultimate internal pressure, the principal tensile strain value of the steel liner is 0.0177. Comparing the principal tensile strain value of the steel liner of the CFRP containment at this internal pressure, it can be obtained that the average reduction for the case of 2200 MPa CFRP tendons is 39.03%, the average reduction for the case of 2400 MPa CFRP tendons is 48.06%, and the average reduction for the case of 2600 MPa CFRP tendons is 82.44%. It can be concluded that the increase in the yield strength of the prestressing tendons can effectively delay the yielding time for the steel liner.

**Figure 6:**
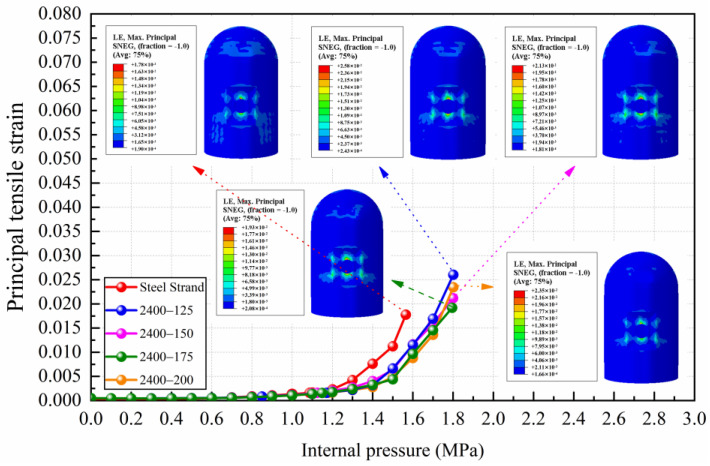
Comparison of principal tensile strain of steel liner for conventional steel strands and CFRP tendons with different elastic moduli.

Figure 6 presents a comparison of the principal tensile strain of steel liner for conventional steel strands and CFRP tendons with different elastic moduli. As shown in Figure 6, when the yield strength of CFRP tendons is the same, the principal tensile strain curves of the containment with different elastic moduli of CFRP tendons almost coincide with each other. It is noticeable that the principal tensile strain curve of the conventional containment deviates from the principal tensile strain curve of the CFRP containment at an internal pressure of 1.2 MPa, which indicates that the elastic modulus has little influence on the yield of the steel liner.

### 4.3. Deformation and Failure Mode

**Figure 7:**
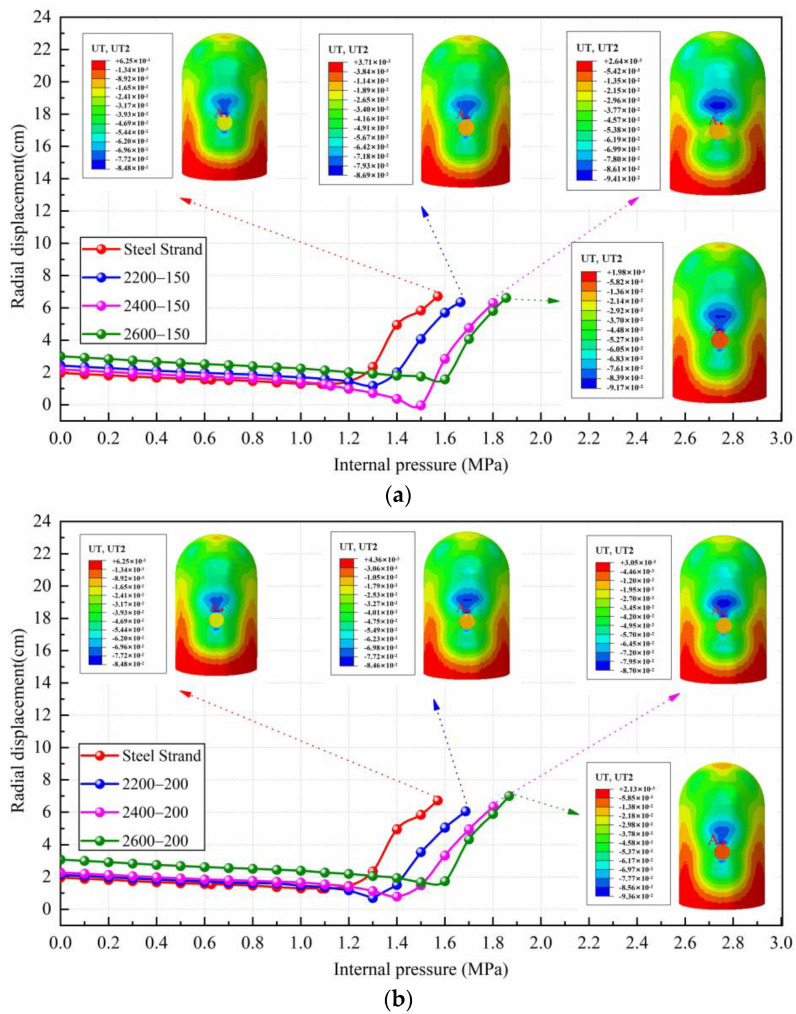
The results of A-point radial displacement of containment with conventional and CFRP tendons: (**a**) comparison of steel strand and CFRP tendons with 150 GPa elastic modulus and different yield strength levels; (**b**) comparison of steel strand and CFRP tendons with 200 GPa elastic modulus and different yield strength levels. Note: point A denotes the top of the equipment hatch.

To facilitate the study of the displacement deformation of the containment, this paper takes points at the top of the equipment hatch, at the top of the dome, and at the side of the containment, denoted as A, B, and C, respectively, to record the displacement change of the containment. Figure 7 draws a comparison of radial displacement at point A of containment with conventional and CFRP tendons possessing different yield strength levels and elastic moduli. As can be seen, the radial displacement at point A for both conventional and CFRP containments is about 6.7 cm when the ultimate internal pressure is reached, and there is no significant change. The main difference is that there is an obvious increase in internal pressure when the maximum value of displacement is reached in the case of adopting CFRP tendons. The higher the strength grade of CFRP tendons used, the more obvious this trend is. It is also noted that the displacement value of point A for the CFRP containment in the elastic phase is slightly larger than that of the conventional containment. For example, under the internal pressure of 1.565 MPa, the radial displacement of point A for 2200 MPa CFRP containments is reduced to 5.26 cm and 4.58 cm for the elastic modulus of 150 GPa and 200 GPa, respectively, which is decreased by 21.6% and 31.7% compared with the conventional containment. The 2400 MPa and 2600 MPa CFRP containments show a similar trend compared with the 2200 MPa CFRP containments.

**Figure 8:**
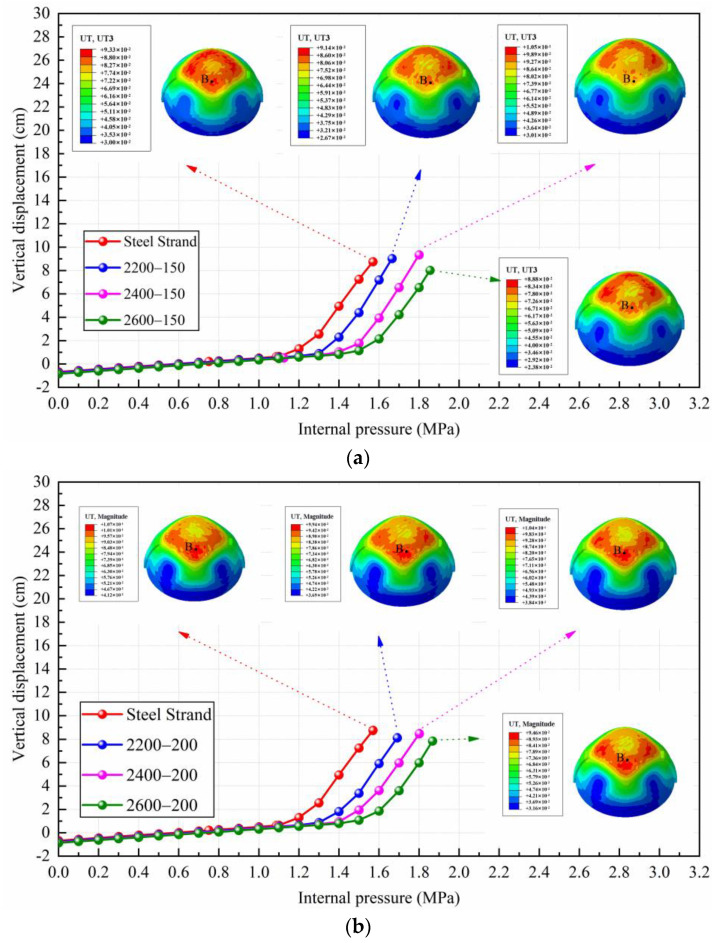
The results of B-point vertical displacement of containment with conventional and CFRP tendons: (**a**) comparison of steel strand and CFRP tendons with 150 GPa elastic modulus and different yield strength levels; (**b**) comparison of steel strand and CFRP tendons with 200 GPa elastic modulus and different yield strength levels. Note: point B denotes the position having the most severe damage for the semicircular dome.

Figure 8 gives the vertical displacement of point B for containments with conventional steel strands and CFRP tendons. From Figure 8, the changing tendency of vertical displacement at point B after adopting CFRP tendons is similar to that of radial displacement at point A. When the conventional containment reaches the ultimate internal pressure, the displacement value of the dome is 8.75 cm. Under the same internal pressure, the vertical displacement values of 2200 MPa, 2400 MPa, and 2600 MPa CFRP containments reduce to 6.19 cm (150 GPa) and 5.04 cm (200 GPa), 3.01 cm (150 GPa) and 3.94 cm (200 GPa), and 1.63 cm (150 GPa) and 1.55 cm (200 GPa), respectively.

**Figure 9:**
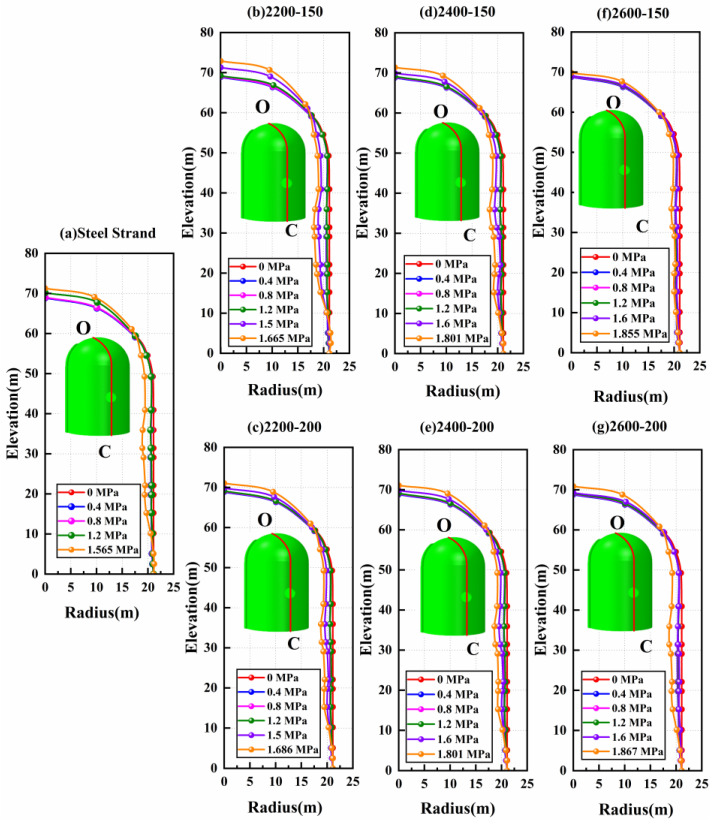
Displacement contour at the centerline of the equipment hatch for conventional and CFRP containments: (**a**) steel strand; (**b**) CFRP tendons with 2200 MPa yield strength and 150 GPa elastic modulus; (**c**) CFRP tendons with 2200 MPa yield strength and 200 GPa elastic modulus; (**d**) CFRP tendons with 2400 MPa yield strength and 150 GPa elastic modulus; (**e**) CFRP tendons with 2400 MPa yield strength and 200 GPa elastic modulus; (**f**) CFRP tendons with 2600 MPa yield strength and 150 GPa elastic modulus; (**g**) CFRP tendons with 2600 MPa yield strength and 200 GPa elastic modulus. Note: points O and C denote the top and bottom of the centerline of the equipment hatch.

Figure 9 plots the displacement contour at the centerline of the equipment hatch for both conventional and CFRP containments. The damage patterns for both conventional and CFRP containments are similar, as both containment domes and cylinders expand outward while the equipment hatch contracts inward under increasing internal pressure. It is noteworthy that the displacement of CFRP containment is significantly smaller than that of conventional containment at the same internal pressure. In addition, for the cases of containment with CFRP tendons possessing the same yield strength but different elastic moduli, it is seen that there is no significant difference for the displacement of points A, B, and C under the same internal pressure, demonstrating that the elastic modulus of the CFRP tendons has little effect on the displacement variation of the containment.

### 4.4. Maximum Principal Stress of Prestressing Tendons

**Figure 10:**
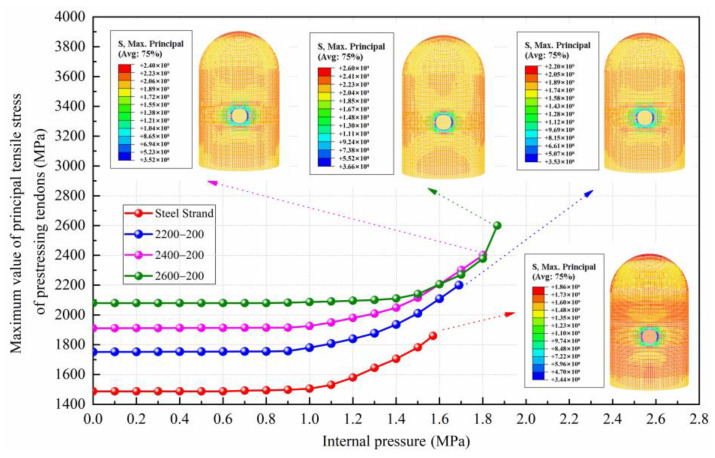
Comparison of principal tensile stresses of prestressing tendons in conventional and CFRP containments.

The maximum principal tensile stress values of prestressing tendons for conventional and CFRP containments are illustrated in Figure 10. The control stress of steel strands is set at 0.8 $f_{\mathrm{ptk}}$ ($f_{\mathrm{ptk}}$ is the ultimate tensile strength of prestressing tendons), and the tensile control stress of CFRP tendons is determined at 0.65 $f_{\mathrm{ptk}}$ (according to Chinese code GB 50608-2010 [37]). When the internal pressure value is 0, the initial principal tensile stress of conventional prestressing tendons is 1488 MPa, while the initial principal tensile stresses of 2200 MPa, 2400 MPa, and 2600 MPa CFRP tendons are 1760 MPa, 1920 MPa, and 2080 MPa, respectively. Compared with conventional containment, the control stress of CFRP containment is greatly increased by 18.28%, 29.03%, and 39.78%, respectively. Because the CFRP tendons have greater ultimate tensile strength, the maximum pressure capacity for CFRP containments would experience a significant increase, with the values increasing to 1.686 MPa, 1.801 MPa, and 1.867 MPa for 2200 MPa, 2400 MPa, and 2600 MPa CFRP tendons, respectively. The ultimate pressure capacity values are 7.7%, 15.08%, and 19.3% higher than those of conventional containment, respectively.

## 5. Conclusions

In this paper, the damage pattern of containments with CFRP tendons under internal pressure is studied and compared with conventional containments. The effects of yield strength and elastic modulus of CFRP tendons on containment are considered comprehensively, and the optimal range of application of CFRP tendons is derived. The following conclusions can be drawn:

1. The crack evolution law of the containment concrete after the replacement of CFRP tendons is basically the same as that of the conventional containment, and the equipment hatch is the first to be damaged, followed by X-shaped cracks. With the increase in internal pressure, cracks appear at the top and bottom. After the internal pressure reaches 1.5 MPa, the cracks increase sharply, and the inverted V-shaped cracks appear at the equipment hatch and gradually deepen. The damage below the equipment hatch is relatively serious.
2. With the increase in the yield strength of CFRP tendons, the crack penetration value at the equipment hatch presents a trend of increasing first and then decreasing. When the yield strength is 2400 MPa, the effect of improving the crack-resistant pressure capacity at the equipment hatch is the best, which is 43.01% higher than that of conventional containment. However, once the yield strength of CFRP tendons exceeds 2400 MPa, the effect of improvement decreases. More seriously, the CFRP tendons with a yield strength of 2800 MPa may induce the crushing of concrete due to excessive prestress.
3. The effect on the steel liner after the use of CFRP tendons is significant, and the internal pressure capacity corresponding to the yield state of the steel liner increases with the increase in the yield strength of CFRP tendons. This is particularly evident for the 2600 MPa CFRP containment, where the internal pressure value corresponding to the yield state of the steel liner has reached 1.348 MPa, a 25.57% improvement over conventional containment. In addition, the maximum principal tensile strain in the steel liner of the 2600 MPa CFRP containment is reduced by 82.44% on average for the same internal pressure.
4. By comparison, it is found that the displacement deformation at points A, B, and C of CFRP containment is smaller than that of conventional containment before reaching the ultimate internal pressure, and the equipment hatch profile curve is smoother. Moreover, the initial control stress of CFRP containment increases, and its ultimate pressure-bearing capacity increases significantly. This indicates that the increase in CFRP prestressing level will increase the initial stiffness of the containment and improve the crack resistance of the containment, which can effectively retard the damage to the containment and reduce the residual deformation.

In a word, the effect of yield strength for CFRP tendons on the concrete cracking, steel liner yielding, and overall displacement deformation of the containment is significant, which substantially improves its working performance under internal pressure. On the contrary, the modulus of elasticity of CFRP tendons does not affect the performance of the containment significantly, and the damage modes of the containment adopting the same grade of CFRP tendons with different moduli of elasticity are almost the same under the comparison of several performance indicators. Finally, this paper determines the best performance improvement effect using CFRP tendons with yield strength ranging from 2200 MPa to 2600 MPa. Considering that the performance improvement effect has started to decline when adopting 2600 MPa CFRP tendons, the 2400 MPa CFRP tendons become the best choice after a comprehensive consideration. Although the research in the paper is concentrated on nuclear containments with CFRP tendons under internal pressure at present, other severe accidents, such as the loss-of-coolant accident, are expected to be investigated to increase the universality of the proposed structure scheme.

## Figures and Tables

**Table 1:** Material properties of concrete.

Concrete Grade	Density (kg/m^3^)	Poisson’s Ratio	Compressive Strength (MPa)	Tensile Strength (MPa)	Modulus of Elasticity (MPa)
C50	2500	0.2	32.4	2.64	34,500

**Table 2:** Material properties of steel and fiber materials [37,38,39].

	Rebar	Steel Strand	Steel Liner	GFRP	AFRP	CFRP
Density (kg/m^3^)	7.85	7.85	7.85	1.25~2.1	1.25~1.4	1.5~1.6
Ultimate tensile strength (MPa)	490~700	1400~1890	/	480~1600	1200~2550	600~3700
Yield strength (MPa)	280~420	1050~1400	320	/	/	/
Modulus of elasticity (GPa)	210	180~200	200	35~65	40~125	120~580
Ultimate elongation	>10.0	>4.0	/	1.2~3.1	1.9~4.4	0.5~1.7
Longitudinal, αL	11.7	11.7	/	8.0~10.0	−6.0~2.0	0.6~1.0
Transverse, αT	11.7	11.7	/	23	30	25
Stress relaxation rate	/	3	/	5	7~20	1~3

**Table 3:** Group number for steel strand and CFRP tendons.

Number	Ultimate Tensile Strength (MPa)	Yield Strength (MPa)	Modulus of Elasticity (GPa)	Number	Ultimate Tensile Strength (MPa)	Yield Strength (MPa)	Modulus of Elasticity (GPa)
A	1860	/	200				
B-1	2500	2000	125	B-2	2500	2000	150
B-3	2500	2000	175	B-4	2500	2000	200
C-1	2750	2200	125	C-2	2750	2200	150
C-3	2750	2200	175	C-4	2750	2200	200
D-1	3000	2400	125	D-2	3000	2400	150
D-3	3000	2400	175	D-4	3000	2400	200
E-1	3250	2600	125	E-2	3250	2600	150
E-3	3250	2600	175	E-4	3250	2600	200
F-1	3500	2800	125	F-2	3500	2800	150
F-3	3500	2800	175	F-4	3500	2800	200

**Table 4:** Statistical information on prestressing loss of steel strands and CFRP tendons.

Tendons Type	Prestress Loss			
	Circumferential	Vertical	Average	Residual Stress (MPa)
A	35.76%	25.97%	30.87%	1028.73
B-1	33.84%	17.87%	25.85%	1186.36
B-2	34.90%	19.04%	26.97%	1168.50
B-3	35.71%	19.92%	27.82%	1154.96
B-4	36.55%	20.82%	28.69%	1141.02
C-1	33.35%	17.28%	25.31%	1314.49
C-2	34.27%	18.30%	26.28%	1297.40
C-3	35.04%	19.13%	27.08%	1283.36
C-4	35.87%	20.02%	27.94%	1268.20
D-1	32.93%	16.77%	24.85%	1442.88
D-2	33.85%	17.80%	25.82%	1424.20
D-3	35.02%	19.10%	27.06%	1400.41
D-4	35.87%	20.01%	27.94%	1383.53
E-1	32.78%	16.59%	24.69%	1566.49
E-2	33.50%	17.38%	25.44%	1550.83
E-3	34.17%	18.12%	26.15%	1536.17
E-4	34.79%	18.81%	26.80%	1522.60
F-1	32.26%	16.25%	24.26%	1711.84
F-2	32.94%	16.99%	24.97%	1695.79
F-3	33.58%	17.61%	25.59%	1681.67
F-4	34.24%	18.23%	26.24%	1666.98

**Table 5:** Statistical information on the sizes of steel strands and CFRP tendons.

	Standard	Single Diameter	Section Diameter	Section Area
Steel strand	1 × 7	5 mm	15.24 mm	140 mm^2^
CFRP tendons	1 × 7	7 mm	21.60 mm	285 mm^2^

**Table 6:** Crack evolution of concrete for conventional and CFRP containments.

Type	0.4 MPa	0.8 MPa	1.1 MPa	1.5 MPa	Ultimate Pressure
A	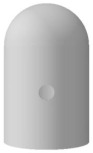	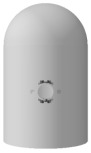	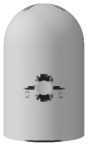	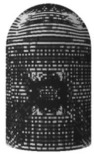	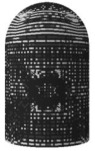
C-2	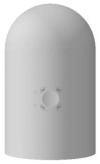	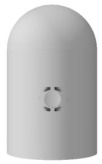	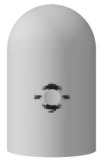	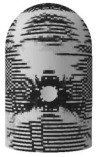	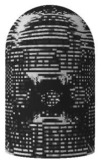
C-4	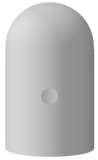	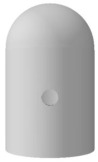	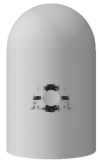	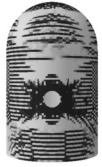	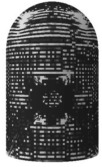
D-2	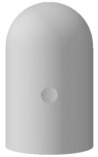	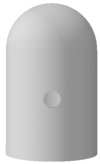	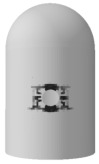	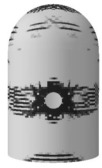	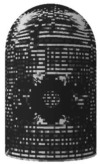
D-4	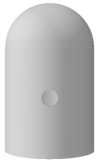	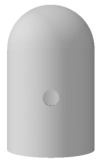	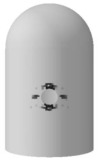	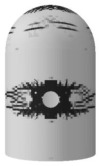	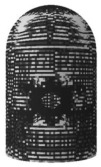
E-2	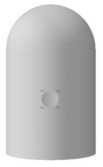	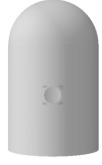	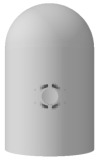	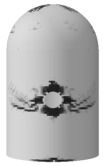	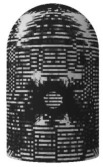
E-4	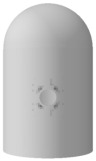	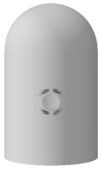	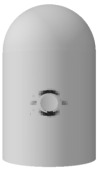	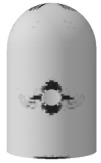	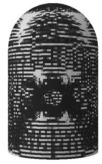

## Data Availability

The data is available after request.
